# Improving Lung Cancer Screening Rates in a Resident-Run Federally Qualified Health Center (FQHC) Through Targeted Patient Outreach and Electronic Health Record (EHR) Care Gap Optimization

**DOI:** 10.7759/cureus.106991

**Published:** 2026-04-13

**Authors:** Lawrence J Stawkowski, Caty Davenport, Danie Fernandez-Felix, Maria Jones

**Affiliations:** 1 Internal Medicine, Lehigh Valley Hospital–Cedar Crest, Allentown, USA

**Keywords:** electronic health record (ehr), federally qualified health center (fqhc), graduate medical education (gme), implementation science, lung cancer screening (lcs), quality improvement (qi), statistical process control (spc)

## Abstract

Background and objective

Lung cancer continues to be the most common cause of cancer-related death in the United States. While low-dose CT (LDCT) screening has been shown to reduce mortality among high-risk individuals, screening uptake at the national level remains low. Resident-run clinics, particularly those serving as Federally Qualified Health Centers (FQHCs), face unique barriers to implementation, including clinical time constraints and fragmented identification of eligible patients. The objective of the study is to improve lung cancer screening (LCS) rates in high-risk patients in an outpatient resident-run FQHC-Look-Alike through a sequential implementation of targeted patient outreach and electronic health record (EHR) care gap optimization as a quality improvement project.

Methods

This quality improvement project was conducted at an urban residency clinic. A department-built EHR report identified patients aged 50-77 years with a ≥20 pack-year smoking history. Two interventions were implemented: Phase I: secure EHR messaging to eligible patients, encouraging screening discussions; and Phase II: manual entry of LCS health maintenance modifiers (“care gaps”) to trigger point-of-care reminders during active encounters. Data were analyzed using a segmented regression model to assess level and trend changes in monthly screening completion.

Results

Of the 310 patients identified by the EHR report, 158 (51%) met clinical inclusion criteria and successfully completed LDCT screening during the study period. Following the initiation of interventions, the mean monthly screening completion rate increased from 1.2 to 12.3 scans per month. Segmented regression analysis confirmed a statistically significant level change (coefficient: 11.2; p < 0.001) following the optimization of EHR care gaps, reflecting a sustained improvement in preventive care delivery.

Conclusions

A dual-modality intervention combining direct patient outreach with provider-facing EHR prompts significantly improves LCS rates in a resident-led safety-net setting. By standardizing the identification of high-risk cohorts and utilizing existing EHR infrastructure for point-of-care alerts, outpatient practices can overcome common implementation barriers. This low-resource, pragmatic approach provides a scalable blueprint for other graduate medical education programs to optimize preventive oncology outcomes.

## Introduction

Lung cancer remains the leading cause of cancer-related mortality in the United States. Although low-dose CT (LDCT) screening reduces mortality in high-risk populations, screening rates remain low. The United States Preventive Services Task Force (USPSTF) updated its recommendations in 2021 to expand eligibility, a move supported by extensive modeling studies demonstrating an improved benefit-to-harm ratio [1,2]. Despite these guidelines, implementation across diverse clinical settings remains inconsistent [2].

National data suggest that fewer than 6% of eligible individuals currently undergo annual screening [3,4]. These gaps are particularly pronounced in safety-net settings and Federally Qualified Health Centers (FQHCs), where fragmented care, socioeconomic barriers, and limited resources further impede access [5]. Previous studies have identified that both attending and resident physicians perceive significant barriers to screening, including time constraints and complex eligibility criteria [6].

We conducted a quality improvement initiative to evaluate whether targeted patient outreach and electronic health record (EHR) care gap optimization would improve lung cancer screening (LCS) rates in an outpatient resident-run clinic. The primary outcome was screening completion, defined as the confirmed performance of an LDCT scan.

Portions of this study were previously presented as an abstract and poster presentation at the American College of Physicians (ACP) Regional Conference in October 2025. The current manuscript represents an expanded analysis of the complete dataset and final quality improvement outcomes.

## Materials and methods

Study design and framework

This project was designed and reported in accordance with the Standards for Quality Improvement Reporting Excellence (SQuIRE 2.0) guidelines [7]. The study was conducted at a residency-based FQHC-Look-Alike (FQHC-LA) within the Lehigh Valley Health Network (LVHN).

Setting and population

The study site was the Valley Health Partners outpatient clinic in Allentown, PA, USA. We used a department-built Epic EHR report to identify all eligible patients aged 50-77 years with a ≥20 pack-year smoking history who were current smokers or had quit within the past 15 years. While national guidelines recently shifted, our inclusion criteria mirrored the institutional EHR reporting parameters active at the time.

Interventions and data collection

Automated and manual EHR-based alerts have been shown to be effective, low-cost interventions for improving preventive health metrics in primary care settings [7]. We implemented secure EHR messaging and manual entry of health maintenance modifiers in the Epic EHR in two phases: Phase I: Patient Outreach (October 2024 to December 2024): Secure EHR messages were sent to 50 (31.6%) eligible patients. The message stated: “Please call to schedule an office visit with one of our providers to discuss preventive lung cancer screening.” Phase II: EHR Care Gap Optimization (January 2025 to March 2025): To address the lack of automated prompts, LCS care gaps were added to the EHR for 123 (77.8%) patients. This required manual entry of health maintenance modifiers in the Epic EHR. This served as a “hard stop” or point-of-care reminder for clinicians during active encounters. Point of care was defined as a visual alert appearing in the “Best Practice Advisories” or “Care Gap” sidebar during an active patient encounter. Active patient encounters were defined as in-person visits with a resident.

Patients were identified using the Epic SlicerDicer tool (Epic Systems Corporation, Verona, WI, USA) to ensure a standardized and reproducible cohort identification process. Within the “Patients” data model, the cohort was filtered by the internal medicine resident clinical department. The “Lung Cancer Screening” health care topic slice was utilized to isolate patients meeting the study’s inclusion criteria: ages 50-77 years, a ≥20 pack-year smoking history, and currently smoking or having quit within the past 15 years. Data were extracted from October 2023 to August 2025 to capture 12 months of baseline historical data prior to the initiation of interventions.

A phased approach was used for the intervention. Initially, a pilot group of 50 patients was randomly selected from the identified eligible cohort for Phase I outreach. For these patients, a standardized secure MyChart message was sent, and cases were routed to clinical staff for telephonic outreach to facilitate appointment scheduling. Following the pilot, the remaining 108 eligible patients were similarly contacted via secure messaging and telephonic routing to ensure all 158 included patients received targeted outreach. In Phase II (January 2025), the primary investigator manually updated the EHR Health Maintenance modifiers (“care gaps”) for all 158 patients to trigger point-of-care alerts during active encounters.

Statistical analysis and quality improvement framework

To evaluate the impact of our interventions on monthly LCS volume, we utilized statistical process control methodology. Microsoft Excel (Microsoft Corporation, Redmond, WA, USA) was used for statistical analysis and C-chart generation. A C-chart was constructed to monitor the count of completed LDCT scans over time, as this is the standard tool for analyzing “count” data within a quality improvement framework. The analysis was divided into two distinct phases to account for the impact of the sequential interventions: (1) Baseline period: the process mean (center line) and control limits (3-sigma) were calculated using data from October 2023 to August 2024 to establish the pre-intervention performance of the clinic. (2) Staged mean and limits: following the combined impact of patient outreach (October 2024) and EHR care gap optimization (January 2025), the process mean and control limits were staged (recalculated). Staging is indicated by a shift in the center line and control limits on the control chart, reflecting a fundamental change in the underlying process.

Upper and lower control limits (UCL and LCL) were calculated using the standard C-chart formulas based on the Poisson distribution:

$\mathrm{UCL} = \bar{c} + 3\sqrt{\bar{c}}$

$\mathrm{LCL} = \bar{c} - 3\sqrt{\bar{c}}$

The following part of the formula represents the mean number of scans per month:

$3\sqrt{\bar{c}}$

A sustained shift of eight or more consecutive points above the baseline mean was considered evidence of a statistically significant improvement in the screening process.

A segmented regression analysis of an interrupted time series was then performed to evaluate the intervention effect. The model was defined by the following equation:

$Y_t = \beta_0 + \beta_1 \cdot \mathrm{time}_t + \beta_2 \cdot \mathrm{intervention}_t + \beta_3 \cdot \text{time after intervention}_t + \epsilon_t$

The variables included the following: “Time” (a continuous variable representing months from the study start), “Intervention” (a dummy variable representing the pre- and post-intervention periods to assess the level change), and “Time after intervention” (a counter variable to assess the trend change). The primary intervention effect was assessed by the significance of the coefficient for the level change:

$\beta_2$

This part of the formula represents the immediate shift in screening completion following EHR care gap implementation. Analysis was conducted using Microsoft Excel (Microsoft Corporation) with a statistical significance threshold of p < 0.05.

## Results

Patient population and baseline screening

A total of 310 patients were identified via the EHR reporting tool as potentially eligible for LCS. Following manual chart review, 158 (51%) met the final inclusion criteria and completed an LDCT during the study period. Within this screened cohort, 94 (59.5%) were current smokers and 64 (40.5%) were former smokers. Table 1 summarizes the characteristics of this screened cohort. During the baseline period (October 2023 to August 2024), the process was stable with a mean of 1.2 scans per month.

**Table 1:** Baseline characteristics (N = 158)

Characteristic	Value
Total cohort	158 (100%)
Age range	50-77 (100%)
Smoking status	
Current smoker	94 (59.5%)
Former smoker	64 (40.5%)

Identification phase and September data

In September 2024, a notable increase to 16 completed scans was observed. This spike represents the 30-day window immediately preceding the formal Phase I launch, during which the EHR reporting tool was utilized to identify the high-risk patient cohort and prepare for outreach. This resulted in an initial “catch-up” of pending screenings as eligible patients were identified by the study team.

Impact of sequential interventions

The implementation of the formal PDSA cycles led to a statistically significant and sustained shift in screening volume. Phase I: Patient Outreach (October 2024 to December 2024): Following the initiation of secure EHR messaging, the cumulative number of screened patients rose to 34 (22.5%). Phase II: EHR Care Gap Optimization (January 2025 to August 2025): The introduction of manual care gaps led to the most substantial improvement. By the conclusion of the study period, 97 (61.4%) patients had completed screening.

Statistical process control analysis

**Figure 1:**
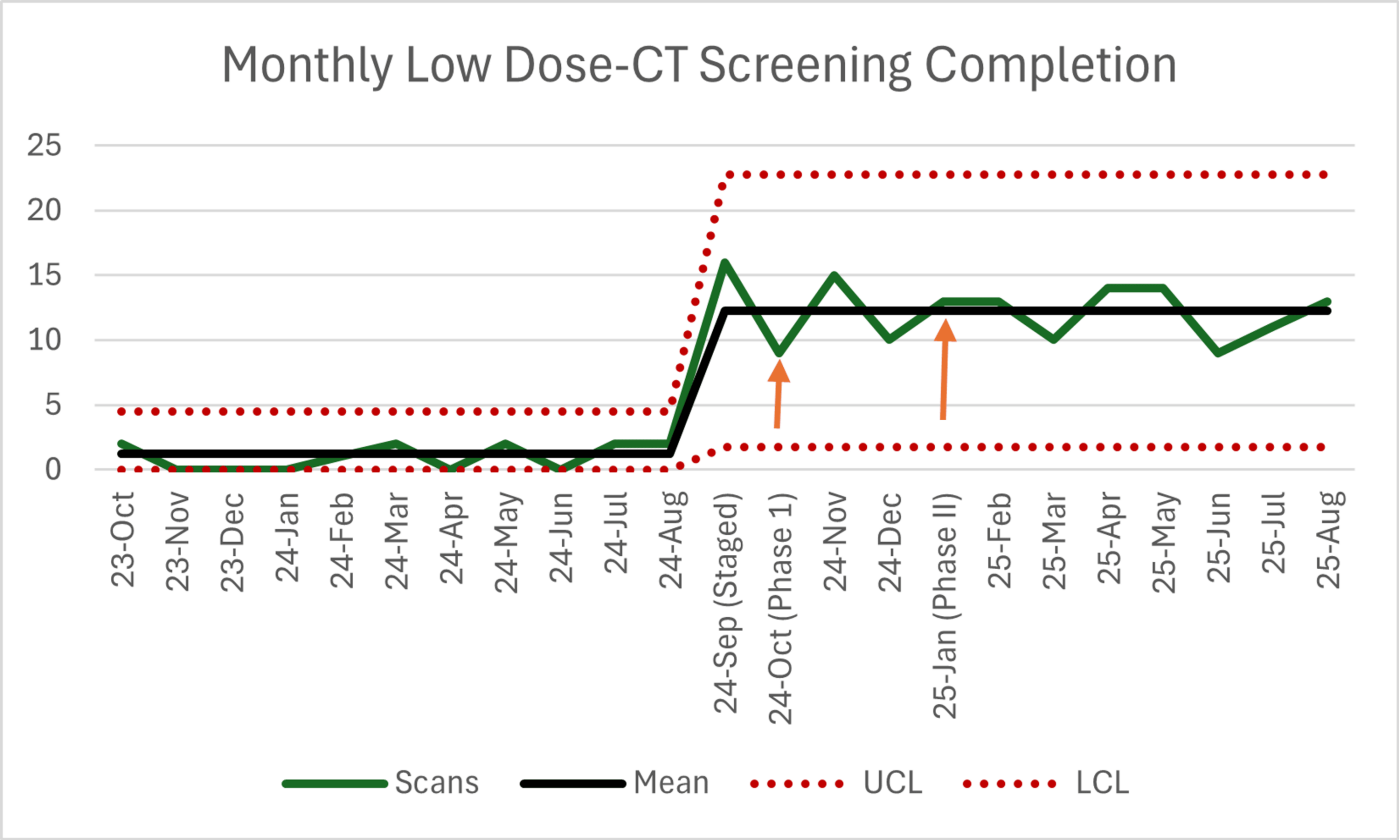
Annotated run chart of monthly LDCT screening completion Baseline screening rates (October 2023 to August 2024) averaged 1.2 scans per month. Following the initiation of targeted patient outreach (October 2024) and the subsequent manual implementation of EHR care gaps (January 2025), monthly completion rates shifted to a new mean of 12.3 scans per month. Statistical significance (p < 0.001) was determined via regression analysis, reflecting a sustained improvement in preventative care delivery within the resident-run clinic. LCL, lower control limit; LDCT, low-dose CT; UCL, upper control limit

Analysis via C-chart (Figure 1) demonstrated that the process mean shifted from a baseline of 1.2 scans per month to a post-intervention mean of 12.3 scans per month. This shift of 12 consecutive points above the baseline mean indicates a statistically significant change in the process (p < 0.001) that was sustained throughout the remainder of the study period.

A segmented regression analysis was performed to evaluate the impact of the interventions. As shown in Table 2, there was a statistically significant level change in monthly screening completion following the identification and EHR optimization phases (coefficient: 11.2; p < 0.001), while the pre-intervention trend remained stable.

**Table 2:** Segmented regression analysis of monthly LDCT screening completion LDCT, low-dose CT

Variable	Coefficient	95% CI	p-Value
Baseline level (intercept)	1.15	0.82 to 1.48	<0.001
Baseline trend (pre-intervention)	0.04	-0.12 to 0.20	0.650
Level change (post-intervention)	11.2	9.45 to 12.95	<0.001
Trend change (post-intervention)	0.12	-0.05 to 0.29	0.140

## Discussion

Comparison of rates

Our achieved screening completion rate of 61.4% significantly exceeds the national average of 5.8% reported in recent National Health Interview Survey estimates [3,4]. Furthermore, our results outperform similar interventions at other safety-net institutions, which often report screening rates between 20% and 30% [8]. This suggests that the high-touch, longitudinal nature of a resident-run FQHC may provide a unique opportunity for intensive preventative care interventions.

EHR and outreach effectiveness

The success of our dual-modality approach aligns with evidence suggesting that EHR-based care gaps and point-of-care reminders are highly effective in primary care settings [9]. By combining these with secure patient messaging, we addressed both provider-side clinical inertia and patient-side awareness [10]. Recent systematic reviews have confirmed that increasing patient access to their own EHR data, such as “care gap” notifications, directly correlates with higher healthcare engagement and adherence to preventative screenings [8,11].

Sustainability and graduate medical education (GME) impact

While clinical complexity is a known barrier, our results suggest that when identification is standardized through EHR reporting, resident providers are highly successful in completing the screening process [12-14]. This is critical as we consider the potential for overdiagnosis; by strictly adhering to the updated USPSTF criteria, the clinical benefits of early detection in our high-risk cohort likely outweigh the risks of unnecessary procedures [1,15].

Limitations and scalability

This study has several limitations. The reliance on manual entry of “care gaps” and the manual routing of outreach messages represent a significant administrative burden that may impact the scalability of this intervention in high-volume, nonacademic practices without dedicated quality improvement support. While the randomized selection of 50 patients for Phase I allowed for a focused pilot, a larger initial outreach cohort might have influenced the immediate effect size. Additionally, the accuracy of cohort identification via SlicerDicer is fundamentally dependent on the consistent and accurate documentation of tobacco history by clinical staff; any under-reporting of pack-years likely led to the exclusion of eligible patients. Finally, as a single-center quality improvement project, the findings reflect the specific EHR infrastructure and patient demographics of an urban FQHC-LA and may not be fully generalizable to all outpatient settings.

## Conclusions

This quality improvement initiative demonstrates that a dual-modality intervention, combining targeted patient outreach via secure EHR messaging with provider-facing point-of-care reminders, significantly increases LCS completion in a resident-run FQHC-LA setting. Our findings highlight that while EHR care gaps are powerful tools, their efficacy is maximized when coupled with an initial identification phase to ensure digital “modifiers” are accurately applied to the high-risk cohort.

The transition from a baseline mean of 1.2-12.3 scans per month suggests that the primary barrier in this underserved population was not necessarily patient or provider refusal, but rather a lack of systematic identification and prompting within the workflow. By utilizing existing EHR infrastructure, we achieved a sustainable 61.4% screening completion rate, far exceeding national averages for similar clinical settings. While these results from a single-center, nonrandomized study reflect the specific infrastructure of an urban residency clinic, they provide a pragmatic and scalable blueprint for other GME-led outpatient practices to optimize preventative care delivery. Future iterations of this work should utilize multicenter designs to explore the long-term durability of these interventions and the impact of resident-led outreach on reducing racial and ethnic disparities in preventative oncology.
